# Concomitant infections of *Plasmodium falciparum *and *Wuchereria bancrofti *on the Kenyan coast

**DOI:** 10.1186/1475-2883-5-8

**Published:** 2006-05-24

**Authors:** Ephantus J Muturi, Charles M Mbogo, Joseph M Mwangangi, Zipporah W Ng'ang'a, Ephantus W Kabiru, Charles Mwandawiro, John C Beier

**Affiliations:** 1 Centre for Ecological Entomology, Illinois Natural History Survey, 607E, Peabody drive Champaign Illinois 61820, USA; 2 Centre for Geographic Medicine Research-Coast, Kenya Medical Research Institute, P.O Box 428, Kilifi, Kenya; 3 Department of Zoology, Kenyatta University, P.O Box 43844, Nairobi, Kenya; 4 Centre for Microbiology Research, Kenya Medical Research Institute, P.O Box 54840, Nairobi, Kenya; 5 University of Miami, School of Medicine, Department of Epidemiology and Public Health. Highland Professional Building, 1801 NW^9th ^Avenue. Suite 300(D-93) Miami, FL 33136 USA

## Abstract

**Background:**

*Anopheles gambiae s.l*. and *An. funestus *are important vectors of malaria and bancroftian filariasis, which occur as co-endemic infections along the Kenyan Coast. However, little is known about the occurrence and prevalence of concomitant infections of the two diseases in mosquito and human populations in these areas. This study reports the prevalence of concomitant infections of *Plasmodium falciparum *and *Wuchereria bancrofti *in mosquito and human populations in Jilore and Shakahola villages in Malindi, Kenya.

**Methods:**

Mosquitoes were sampled inside houses by pyrethrum spray sheet collection (PSC) while blood samples were collected by finger prick technique at the end of entomological survey.

**Results:**

A total of 1,979 female *Anopheles *mosquitoes comprising of 1,919 *Anopheles gambiae s.l *and 60 *An. funestus *were collected. Concomitant infections of *P. falciparum *sporozoites and filarial worms occurred in 1.1% and 1.6% of *An. gambiae s.l *collected in Jilore and Shakahola villages respectively. *Wuchereria*-infected mosquitoes had higher sporozoite rates compared to non-infected mosquitoes, but multiple infections appeared to reduce mosquito survivorship making transmission of such infections rare. None of the persons examined in Shakahola (n = 107) had coinfections of the two parasites, whereas in Jilore (n = 94), out of the 4.3% of individuals harbouring both parasites, 1.2% had *P. falciparum *gametocytes and microfilariae and could potentially infect the mosquito with both parasites simultaneously.

**Conclusion:**

Concerted efforts should be made to integrate the control of malaria and bancroftian filariasis in areas where they co-exist.

## Background

Malaria and lymphatic filariasis are the world's most important parasitic infections with an estimated loss of 4.5 × 10^7 ^and 4.9 × 10^6 ^disability adjusted life years (DALYs), respectively [1,2]. The two diseases occur as co-endemic infections in many tropical developing countries affecting the same human hosts and sharing common vectors [3]. In Papua New Guinea, Burkot *et al*. [4] reported the occurrence of concomitant infections of *P. falciparum *and *W. bancrofti *in *An. punctulatus *mosquitoes, whereas in India and South America, the two parasites have been reported to occur simultaneously in humans [5-7].

Several reports indicate that a high prevalence of bancroftian filariasis and falciparum malaria occurs along the Kenyan Coast [8-12]. Moreover, *An. gambiae s.l *and *An. funestus *combine the dual role in their transmission [13-16]. Concomitant infections of the two diseases is therefore expected to be a common feature in both humans and mosquito vectors in these areas but little information exists about the occurrence of this phenomenon. In Tanzania, multiple infections of malarial and filarial parasites were documented five decades ago in humans and *An. gambiae *[17] but little is known about how the two parasites interact during concomitant parasitism. Studies in India have demonstrated that the intensity of *P. falciparum *is generally lower in microfilaraemic individuals than in amicrofilaraemic ones [6]. Moreover, laboratory studies using vertebrate hosts have revealed that filarial infections may have either benign or suppressive effect on malaria development [18,19]. For instance, microfilaraemic infections in owl monkeys, *Aotus trivirgatus griseimembra *resulted in more benign *P. falciparum *infections than in amicrofilaraemic monkeys [18]. This indicates that interactions between malaria and filarial parasites may influence the clinical presentation, pathogenicity, and even epidemiology of the diseases they cause [6], making them important to both the clinicians and epidemiologists.

In mosquito vectors, interaction between pathogens may affect susceptibility of the vector to infection. Under laboratory conditions, Turrell *et al*. [20] demonstrated that *Aedes taerniorhynchus *mosquitoes infected with *Brugia pahangi *were more susceptible to Rift Valley Fever (RVF) Virus. This was attributed to the physical disruption of midgut by migrating microfilariae, allowing penetration of virus into the haemocoel. A similar mechanism was suggested to be responsible for the high number of *W. bancrofti *larvae observed in *Plasmodium*-infected *An. punctulatus *[4]. In contrast however, heavier and/or mixed malaria and filarial infections affect vector survival and flight behaviour [21-24] resulting to reduced transmission of both parasites simultaneously [17]. It is therefore of epidemiological significance to study how malaria and filarial parasites interact in *An. gambiae s.l *and *An. funestus*, the Africa's most important vectors of malaria and LF. The present study was conducted to investigate the occurrence and prevalence of concomitant infections of *P. falciparum *and *W. bancrofti *in mosquito and human populations along the Kenyan Coast. The results of this study provide important information on the need for integrating the control of malaria and lymphatic filariasis.

## Materials and methods

### Study area

The study was carried out in Shakahola and Jilore villages in Malindi District in Coastal Kenya. The District lies between latitude 2.20 degrees East and 4 degrees South and longitude 3 degrees and 4.14 degrees East. The study area is hot and humid all year round with the annual mean temperatures ranging between 22.5°C and 34°C and the average relative humidity ranging between 60% and 80%. There are two main rainfall seasons in a year. The long rains start from April to June with a peak in May while the short rains fall from October to November. The annual average rainfall ranges from 400 mm in the hinterland to 1200 mm in the Coastal belt. The soils are mainly sandy and infertile supporting small patches of natural forest interpassed with bushes and grasslands.

Jilore Village is located approximately 30 km west of Malindi town and has a population of 543 people. The village borders the extensive Sokoke forest to the south. The site has a hilly terrain with few plateaus and sandy soils. The population is composed of subsistence farmers, growing mainly maize and cassava and keeping chicken, ducks, and goats as domestic animals. Coconuts and cashew nuts are produced for commercial purposes. Small-scale fishing is also practiced in Lake Jilore. The inhabitants are mainly the Giriama, one of the groups making up the nine-mijikenda people of the East Africa coastal region. Majority of them live in mud-walled houses thatched with coconut leaves. The houses have unscreened widows, holes in the walls, and large open eaves that provide easy entry for mosquitoes. Homesteads are scattered and separated from one another either by agricultural land or small patches of natural vegetation. Each homestead has a number of houses, which are as a result of extended families sharing one compound. Domestic water is collected from the permanent Sabaki River, which together with Lake Jilore provide suitable larval habitats for mosquitoes. The population seeks treatment from Jilore Dispensary. In the year 2002 the prevalence of malaria in this dispensary was 40.5% all due to *P. falciparum *[25]. Lymphatic filariasis is also common in the village as depicted by the high number of people with overt symptoms of the disease (Mwandawiro, personal communication). However, it is rarely diagnosed in the dispensary. Historically, there has never been a filariasis control programme in the village, but it is currently underway.

Shakahola village is located approximately 90 km southwest of Malindi town and 45 km from Jilore. The village has a population of 1,009 people. The site is located on a fairly flat terrain with few hilly terrains. The population is composed of subsistence farmers, and essentially they grow the same crops and keep the same domestic animals as those in Jilore. Palm-thatched-mud walled houses, scattered homesteads, and extended families also characterize the village. River Sabaki is the main source of domestic water for the inhabitant. The population is composed of the Kauma and Giriama sub-tribes, which are among the groups making up the nine-mijikenda people of the East Africa coastal region with the Giriama constituting over 75% of the population. The inhabitants seek treatment from the neighboring Chakama Dispensary. Malaria is an important health problem in the area and had a prevalence of 29.5% in the year 2002 all due to *Plasmodium falciparum *[25]. Lymphatic filariasis is also highly endemic although it is rarely diagnosed. Currently there is an ongoing filariasis control programme in the village and by the beginning of this study, all the inhabitants had already taken the first annual single dose combination of diethycarbamazine and albendazole drugs five months earlier. The prevalence of microfilaraemia in humans before treatment was 17.7% while the filarial infection and infectivity rates were 9.4% and 3.0% in *Anopheles gambiae s.l*, and 3.3% and 1.0% in *An. funestus *(Mwandawiro, unpublished report).

### Registration of inhabitants

Before fieldwork commenced, permission to conduct the study was obtained from Kenya Medical Research Institute (KEMRI) ethical review committee. Meetings were held in the villages to explain the purpose of the study to the inhabitants. It was made clear that participation in the study was voluntary and that all members of the household aged four years and above were eligible for enrolment in the study. Before commencing the collection of blood, census of all people was done by house-to-house visits, during which personal information was taken for each individual. Individual consent was obtained from each participant or (if < 16 years) from one of their parents or guardian. Individuals found positive for malaria, filarial or both parasites were advised to seek treatment.

### Mosquito collection

In each village, ten randomly selected houses were sampled for mosquitoes between 0700 and 1000 hours. In Shakahola, mosquitoes were collected in each of the 10 houses once in a month over a three-month period namely; September 2002 and January and February 2003. Due to logistical difficulties sampling was not done between October and December 2002. In Jilore, the collections were done over a 6-month period, sampling each of the ten houses every alternate day for five days in September, three days in October, November and December 2002 and once in January and February 2003. The monthly differences in mosquito sampling times were also as a result of logistical difficulties.

During sampling, pyrethrum spray sheet collection [26] was conducted in five of the ten selected houses per village while the remaining five houses were sprayed the following day. All the knocked down female anopheline mosquitoes from different households were picked up from among other insects on the sheets, placed into labeled petri dishes lined with moist cotton wool and transported to the laboratory in a cool box for identification and dissection.

### Mosquito identification and processing

Mosquitoes were identified morphologically to species using taxonomic keys [27]. The head, thorax and abdomen of each *An gambiae s.l *and *An. funestus *were dissected separately from each other in a drop of phosphate buffered saline (PBS) on a slide and examined for filarial worms [28]. The worms were classified as L_1 _(sausage stage), L_2_(motile short) and L_3 _(motile, infective and with caudal papillae) larvae [29]. The number of larvae was counted to determine the infection load per mosquito.

The debris of the heads and thoraces of all *Anopheles *mosquitoes examined for filarial worms were removed from the slides and placed singly into labeled plastic vials paying much attention to avoid contamination. Eventually, 50 μl of boiled casein blocking buffer with Nonidet 40 were added into each vial, and the samples ground using sterile pestles. Subsequently, 200 μl of blocking buffer were added bringing the final volume to 250 μl. The samples were stored at -20°C until time of testing. Fifty microlitres aliquots were tested by an enzyme-linked immunosorbent assay (ELISA) using monoclonal antibodies to detect circumsporozoite (CS) proteins of *P. falciparum *[30]. Samples were assessed visually for positivity [31].

### Parasitological survey

Blood samples were taken once, from volunteers aged four years and above and living in the two villages. Briefly, 130 μl of finger-prick blood was collected in a heparinized capillary tube between 2100 and 2400 hrs [32]. One hundred microliters of this blood was immediately transferred into a plastic vial containing 0.9 ml of 3% acetic acid. In the laboratory, each specimen was transferred to a clean counting chamber and examined under a microscope for enumeration of *Wuchereria bancrofti *microfilariae [33].

The remaining 30 μl of blood was used to prepare thick and thin smears for examination of malaria parasites. The smears were stained with 10% Giemsa for 10 minutes, and then examined microscopically under oil immersion for *Plasmodium *species [32]. A person was considered to be malaria positive if malaria parasites were detected in the thick blood smear and negative if no parasites were found in 200 fields of the thick smear. The thin smears were used for *Plasmodium *species identification. When malaria parasites were demonstrable in the thick blood film, the parasitaemia (parasite density) was determined by counting the number of parasites per 200 leucocytes and total number of parasites obtained multiplied by 40 based on a mean leukocyte count of 8,000 per microliter of blood [34]. A person was considered to have concomitant infections of malaria and bancroftian filariasis if positive for both malaria and microfilariae parasites.

### Statistical analyses

Data from precoded forms was checked for accuracy and entered into the computer using FoxPro programme. The contents of the computer files were then checked against the original precoded data sheets for errors or omissions. All statistical analyses were performed using SPSS software (Version 11.5 for widows, SPSS Inc., Chicago, IL). The differences in sporozoite rates, filarial infection, and infectivity rates between species and villages as well as the differences in prevalence of microfilaraemia and malaria parasites between the two villages were compared by chi-square or Fisher's exact test (as appropriate). Interaction between malaria and filarial parasites was determined using the student t-test. The geometric mean density of malaria parasites in humans was calculated after logarithm transformation to normalize the distribution and minimize the standard error.

## Results

### Prevalence of *P. falciparum *and *W. bancrofti *in *An. gambiae s.l *and *An. funestus*

Table 1 shows the prevalence of *P. falciparum *and *W. bancrofti *in *An. gambiae s.l *and *An. funestus*. In Jilore, *P. falciparum *sporozoite rates and *W. bancrofti *infection and infectivity rates were 7.7%, 5.9% and 1.1%, respectively in *An. gambiae s.l *and 1.7%, 6.9% and 1.7% in *An. funestus*. These differences in sporozoite rates and filarial infection and infectivity rates between the two species were not significant (Fisher's exact test, p = 0.123, p = 0.775 and p = 0.484). The corresponding sporozoite rates, filarial infection and infectivity rates for *An. gambiae s.l*. in Shakahola were 5.9%, 13.0% and 0.5%, respectively. None of the two *An. funestus *caught was found positive for any of the two parasites. Filarial infection rates in *An. gambiae s.l *were significantly higher in Shakahola than in Jilore (Fisher's exact test, p = 0.01).

**Table 1:** *Plasmodium falciparum *sporozoites rates and filarial infection rates in *An. gambiae s.l *and *An. funestus *in Jilore and Shakahola villages

villages	Mosquito species	Number examined	Sporozoite rates	Filarial infection rates (L_1_-L_3_)	Infectivity rate (L_3_)
Jilore	*An. gambiae s.l*	1734	7.7	5.9	1.1
	*An. funestus*	58	1.7	6.9	1.7
Shakahola	*An. gambiae s.l*	185	5.9	13.0	0.5
	*An. funestus*	2	0.0	0.0	0.0
Total	*An. gambiae s.l*	1919	7.6	6.6	1.0
	*An. funestus*	60	1.7	6.7	1.7

### Concomitant infections of *P. falciparum *and *W. bancrofti *in *An. gambiae s.l*

Concomitant infections of *P. falciparum *sporozoites and *W. bancrofti *larvae were recorded in 1.1% (n = 1,734) and 1.6% (n = 185) of *An. gambiae s.l *from Jilore and Shakahola, respectively (Table 2). Only 10.5% (n = 19) of mosquitoes infected with both parasites were observed harbouring the infective larvae (L_3_) of *W. bancrofti *together with *P. falciparum *sporozoites compared to 89.5% of mosquitoes that harboured *P. falciparum *sporozoites together with immature stages (L_1 _and L_2_) of *W. bancrofti*. None of the 60 *An. funestus *harboured both parasites simultaneously.

**Table 2:** Mixed infections of *P. falciparum sporozoites *and *W. bancrofti *larvae in *An. gambiae s.l*. in Jilore and Shakahola villages

	Number of mosquitoes infected (%)	
Mixed infections	Jilore (n= 1734)	Shakahola (n= 185)

L1 and Sporozoites	11 (0.6)	2 (1.1)
L2 and sporozoites	3 (0.17)	1 (0.54)
L3 and sporozoites	1 (0.06)	0 (0.0)
L1, L2 and sporozoites	3 (0.17)	0 (0.0)
L1, L3 and sporozoites	1 (0.07)	0 (0.0)
L2, L3 and sporozoites	0 (0.0)	0 (0.0)
L1, L2, L3 and sporozoites	0 (0.0)	0 (0.0)

Total	19 (1.1)	3 (1.62)

The *P. falciparum *sporozoite rate in mosquitoes with and without filarial infections is shown in Table 3. *An. gambiae s.l *infected with filarial worms in both villages had higher sporozoites rates than those without filarial infection, although significant difference was only observed in Jilore (χ^2^, p < 0.001).

**Table 3:** *Plasmodium falciparum *infections in mosquitoes with and without filarial worms (all stages) in Jilore and Shakahola villages

Site	Species	Filarial infection	No. of mosquitoes	No. with sporozoite (%)	P value
Jilore	An. gambiae s.l	Non-infected	1631	115 (7.1)	
		Infected	103	19 (18.4)	
	Overall		1734	134 (7.7)	χ^2^, p < 0.001
	
	An. funestus	Non-infected	54	1 (1.9)	
		Infected	4	0 (0.0)	
	Overall		58	1 (1.7)	FET, p = 1.000

Shakahola	An. gambiae s.l	Non-infected	161	8 (5.0)	
		Infected	24	3 (12.5)	
	Overall		185	11 (5.9)	FET, p = 0.157
	
	An. funestus	Non-infected	2	0 (0.0)	
		Infected	0	0 (0.0)	
	Overall		2	0 (0.0)	

FET; Fisher's exact test

### Prevalence of malaria parasites

Table 4 shows the prevalence of malaria parasites in humans. A total of 208 individuals were examined for malaria parasites of which 47.6% (n = 99) were from Jilore and 52.4% (n = 109) from Shakahola. The prevalence of *P. falciparum *was significantly higher in Jilore (36.4%) than in Shakahola (17.4%) (χ^2^, p = 0.002), whereas *P. malariae *was only found in Jilore (1.0%) but not in Shakahola. The geometric mean density of malaria parasites was 726.9 parasites/μl of blood in Shakahola and did not differ significantly from 505.0 parasites/μl of blood in Jilore (t, p = 0.227). The prevalence of *P. falciparum *gametocytes was 4.0% in Jilore whereas in Shakahola none of the persons examined had gametocytes. The geometric mean density of gametocytes in Jilore was 115.7 gametocytes/μl.

**Table 4:** Malaria and microfilariae prevalence among individuals in Jilore and Shakahola villages.

*P. falciparum *prevalence			*P. malariae *prevalence	Gametocyte prevalence	Micofilaraemia	
Village	No. examined	No. positive (%)	No. positive (%)	No. positive (%)	No. examined	No. positive (%)

Jilore	99	36 (36.4)	1 (1.0)	4 (4.0)	94	15 (16.0)
Shakahola	109	19 (17.4)	0 (0)	0 (0)	107	3 (2.8)

Overall	208	55 (26.4)	1 (0.5)	4 (1.9)	201	18 (9.0)

### Prevalence of microfilaraemia

The prevalence of microfilaraemia in Jilore and Shakahola villages is shown in Table 4. Of the 208 individuals examined for malaria parasites, 201 of them were further examined for microfilariae. Overall, Jilore (16.0%) had significantly higher microfilaraemia prevalence compared to Shakahola (2.8%) (χ^2^, p < 0.001).

Microfilaraemia prevalence in both villages increased with age, peaking in the over 50 year's age group where the overall prevalence was 42.9% and 16.7% in Jilore and Shakahola, respectively (data not shown).

### Prevalence of concomitant infections of malaria and bancroftian filariasis in humans

Two hundred and one people from both villages were examined for malaria and microfilariae parasites, out of which 2.0% had both *P. falciparum *and microfilariae parasites. None of the persons examined in Shakahola (n = 107) had mixed malaria and filarial infections whereas in Jilore (n = 94), 4.3% of the individuals examined harboured microfilariae and *P. falciparum*, 1.2% of these carrying both microfilariae and *P. falciparum *gametocytes. All the persons harbouring both parasites were from 10–29 years age group. When the difference in the geometric mean density of *P. falciparum *was compared between microfilaraemic and amicrofilaraemic persons, no significant difference was observed (t, p > 0.05).

## Discussion

Malaria and LF co-exist in human populations along the Kenyan coast and transmitted by common vectors. However, there has been inadequate information on the occurrence and prevalence of concomitant infections of the two diseases in both the vector and human populations in these areas. The World Health Organization is currently implementing a new framework for vector control based on a strategy of integrated vector-management targeting both diseases simultaneously. It is thus deemed essential to obtain local information on the occurrence, distribution and prevalence of co-infections of the two diseases as a first step towards this goal. The main aim of the present study was to collect baseline data on which efforts towards designation and implementation of an integrated control strategy may be based. We therefore considered two sets of villages, one in which filariasis control programme was already in place and another one where filariasis control programme is under way. Due to logistical difficulties, we were unable to replicate each set of village but we felt that the two villages would provide essential ideas on the actual situation on the ground.

In Jilore where no LF control programme was in place, we observed significantly higher sporozoite rates in *Wuchereria*-infected *An. gambiae s.l *than in non-infected mosquitoes. This indicates that infection with *W. bancrofti *may increase mosquito susceptibility to *P. falciparum*. In Papua New Guinea, *Plasmodium*-infected *An. punctulatus *had higher number of W. *bancrofti *larvae compared to uninfected mosquitoes [4]. Mosquito gut is known to be a significant barrier to malaria infection [35], but its physical disruption by migrating microfilariae removes this barrier. This may account for the higher sporozoite rates in *Wuchereria*-infected mosquitoes compared to non-infected mosquitoes. Surprisingly, despite the high sporozoite rates in *Wuchereria*-infected mosquitoes, the results demonstrated that simultaneous transmission of concomitant infections of the two parasites is rare. This is reflected by the absence of concomitant infections in *An. funestus *and the low number of *An. gambiae s.l *that were found harbouring both *P. falciparum *sporozoites and infective larvae of *W. bancrofti*. A similar study along the Kenyan coast reported that only 0.4% of *An. gambiae s.l *harboured the infective stages of malaria and filarial parasites [36]. In Tanzania, only a single mosquito was found harbouring infective stages of malaria and filarial parasites out of 15 mosquitoes that were found infected with malaria or filaria [17]. This suggests that majority of mosquitoes that pick up mixed malarial and filarial infections do not live long enough for the two parasites to reach the infective stage. Under laboratory conditions, a greater proportion of *B. pahangi *failed to complete development to the second stage in *Aedes aegypti *mosquitoes harbouring *Plasmodium gallinaceum *[37]. This confirms previous findings that enhanced susceptibility to multiple infections in mosquitoes is of no advantage to parasite transmission [23]. The findings of this study have important implications towards control of malaria and bancroftian filariasis. Based on the results from Jilore, one may be misled to conclude that elimination of bancroftian filariasis would result to a reduction in sporozoite rates in mosquitoes. However, although further studies are needed to assess the effect of LF control on malaria transmission where both diseases are co-endemic, the findings of this study did not establish any significant difference in *P. falciparum *sporozoite rates between *Wuchereria*-infected and non-infected mosquitoes in Shakahola where there was an ongoing LF control programme. This indicates that differential elimination of LF in malaria endemic areas may result to an increase in malaria transmission since many mosquitoes that die before *W. bancrofti *larvae become infective [38] or due to multiple infections [23] will be spared. This clearly supports the need for integrated control of the two diseases. Currently, the Global Programme for Elimination of Lymphatic Filariasis (GPELF) and the Roll Back Malaria Partnership (RBM) carry out most of vector control. RBM is targeting the *Anopheles *vectors of malaria and its activities also cut filarial transmission, particularly where *Anopheles *are also the main vectors of *W. bancrofti*. Co-ordination of the activities of RBM and GPELF could ensure the reduction of both diseases, thereby offsetting the increase in malaria transmission that could arise from differential elimination of bancroftian filariasis.

Our results indicate that co-infections of malaria and LF in the population are more likely to occur when the prevalence of both diseases is high. This is clearly demonstrated by the presence of co-infections of the two diseases in Jilore and their absence in Shakahola. In coastal areas of Georgetown, Guyana low incidence of malaria was used to explain the low transmission of concomitant infections of malaria and filariasis [7]. Reducing the prevalence of both diseases would thus reduce the prevalence of concomitant infections of the two diseases. Such a goal can be achieved through early diagnosis and treatment of malaria and filariasis. Unlike studies in India and South America [6,7], the present study has demonstrated that some individuals harbour both malaria gametocytes and microfilariae parasites. A mosquito feeding on such individuals may therefore pick both parasites simultaneously. On the other hand, this study has only demonstrated the occurrence of *P. falciparum *together with *W. bancrofti *but not with other *Plasmodium *species like *P. vivax *as has been observed in India [5]. This was expected since *P. falciparum *constituted 99.5% of malaria prevalence while *P. malariae *constituted the remaining 0.5%. Since concurrent transmission of the two parasites by anophelines on the Kenyan Coast is rare [36], and mass treatment of people with DEC and albendazole interrupts their occurrence in humans, as depicted by their absence in Shakahola, promotion of insecticide-treated bed nets together with mass administration of antimalarial and filaricidal drugs can be utilized in control of malaria, bancroftian filariasis and co-infection with the two diseases.

Although this study was limited by the small sample size, concomitant infections of malaria and bancroftian filariasis were only observed in persons aged between 10 and 29 years. Chadee *et al *[7] has also reported the absence of such infections in children below 10 years in South America. The differences in behaviour and occupation of people in different age groups may account for the distribution of concomitant infections. The 10 to 29 years age group is composed of young school going children and energetic working individuals. These children are active and may engage in outdoor activities until late evening exposing themselves to mosquito bites, while the working individuals may be exposed to infective bites while working. The fishing in Lake Jilore, and tapping of palm wine (*Mnazi*) both of which are conducted early in the morning and late in the evening may expose people to mosquitoes. Young children and older adults retire to their homes earlier, but are also at risk since these vectors are known to be highly endophilic and anthropophilic [39].

The present study did not reveal any significant difference between the geometric mean density of *P. falciparum *in microfilaraemic and amicrofilaraemic individuals, an indication that filarial infections do not affect *P. falciparum *parasitaemia. This was not expected since helminthes are known to induce a TH2-dominant response, which may alter cell mediated immune function to other microbial agents [40]. Moreover, studies in India [6] and in animal models [18,19] have demonstrated that filarial infection result in either benign or suppressive effect on *P. falciparum *infections.

## Conclusion

*Wuchereria-*infected mosquitoes seem to be more susceptible to *P. falciparum *infections, but this does not offer any advantage to the transmission of both parasites due to increased mortality of the affected mosquitoes. Thus differential elimination of bancroftian filariasis could potentially increase mosquitoes' life-span resulting to increase in malaria transmission. Furthermore, concomitant infections of malaria and filariasis are likely to occur when the prevalence of both parasites is high. As such, an integrated control strategy targeting the two diseases in areas where they are co-endemic is recommended. Further studies should be conducted under field and laboratory conditions, to ascertain the hypothesis that *Wuchereria *infection increases mosquito susceptibility to *P. falciparum *and appropriate control strategies designed and implemented.

## Competing interests

The author(s) declare that they have no competing interests.

## Authors' contributions

Ephantus J. Muturi conducted the survey and drafted the manuscript. Charles M. Mbogo provided scientific guidance in data collection, analysis and manuscript preparation and planning, and implementation of day-to-day field activities. Joseph M. Mwangangi, Zipporah W. Ng'ang'a and Ephantus W. Kabiru offered scientific guidance in data analysis and manuscript preparation. Charles Mwandawiro assisted in data collection, provision of ethical clearance and guided in data collection, analysis and manuscript preparation. John C. Beier provided overall supervision of the study and preparation of manuscript. All authors read and approved the final manuscript.
